# The medial temporal lobe—conduit of parallel connectivity: a model for attention, memory, and perception

**DOI:** 10.3389/fnint.2014.00086

**Published:** 2014-11-11

**Authors:** Brian Mozaffari

**Affiliations:** Menninger Department of Psychiatry and Behavioral Sciences, Baylor College of MedicineHouston, TX, USA

**Keywords:** perception, memory, attention, free energy, hippocampus, medial temporal lobe, episodic memory

## Abstract

Based on the notion that the brain is equipped with a hierarchical organization, which embodies environmental contingencies across many time scales, this paper suggests that the medial temporal lobe (MTL)—located deep in the hierarchy—serves as a bridge connecting supra- to infra—MTL levels. Bridging the upper and lower regions of the hierarchy provides a parallel architecture that optimizes information flow between upper and lower regions to aid attention, encoding, and processing of quick complex visual phenomenon. Bypassing intermediate hierarchy levels, information conveyed through the MTL “bridge” allows upper levels to make educated predictions about the prevailing context and accordingly select lower representations to increase the efficiency of predictive coding throughout the hierarchy. This selection or activation/deactivation is associated with endogenous attention. In the event that these “bridge” predictions are inaccurate, this architecture enables the rapid encoding of novel contingencies. A review of hierarchical models in relation to memory is provided along with a new theory, Medial-temporal-lobe Conduit for Parallel Connectivity (MCPC). In this scheme, consolidation is considered as a secondary process, occurring after a MTL-bridged connection, which eventually allows upper and lower levels to access each other directly. With repeated reactivations, as contingencies become consolidated, less MTL activity is predicted. Finally, MTL bridging may aid processing transient but structured perceptual events, by allowing communication between upper and lower levels without calling on intermediate levels of representation.

## Introduction

The medial temporal lobe (MTL)—consisting of the hippocampus and adjacent anatomically related cortex, including the enorhinal, perirhinal, and parahippocampal corticies—has been assigned a number of functions; however, a coherent and inclusive functional definition remains elusive. A traditional view holds that the hippocampus forms a unitary memory storage system—along with other MTL structures (Squire and Zola-Morgan, 1991; Squire and Wixted, 2011). Another popular view holds that the hippocampus mediates recollective memory rather than familiarity-based recognition (Eldridge et al., 2000; Diana et al., 2007; Shimamura, 2010; Yonelinas et al., 2010). Other work suggests that MTL activity is not exclusive to memory, but is also engaged during perceptual processing of complex scenes (Eacott et al., 1994; Buckley et al., 2001; Lee et al., 2005; Lech and Suchan, 2013). Overall many different functions have been ascribed to the MTL.

The primary aim of MTL Conduit for Parallel Connectivity (MCPC) is to view MTL functionality through the free energy-hierarchical framework as opposed to a framework where connections between perception, cognition, and memory are not apparent. In order to convey this concept, this paper will describe memory, cognition, perception, and attention like they are separate processes while simultaneously introducing a framework that demonstrates their fluent interconnections.

Medial temporal-lobe Conduit for Parallel Connectivity focuses primarily on memory; however, attention and perception are covered since they are fundamental to hierarchical inference. First, to contextualize the theoretical tenets of MCPC, two pertinent theories of memory will be discussed: Standard Model of Systems Consolidation (SMSC) and Multiple Trace Theory (MTT). Second, a brief review of hierarchical generative models in relation to the free energy principal will be supplied to serve as the foundation of MCPC. Afterwards, the MCPC model is presented followed by a comparison to a similar model, Predictive Interactive Multiple Memory Systems (PIMMS) to further explain nuances of the framework proposed. Finally, an attempt is made to clarify differences between MTT and SMSC within the free energy-hierarchical framework.

## Two pertinent models of hippocampal function

Currently, two major theories make predictions regarding the role of the hippocampus. The first is the SMSC, which holds that the initial memory trace is simultaneously encoded in the cortex and hippocampus (Squire and Alvarez, 1995). It states that the cortex is unable to initially contain the memory alone; however, with support from the hippocampus, appropriate associative connectivity forms within the cortex and the memory is stored. Overall, SMSC predicts that the hippocampus is not required for retrieval of remote memories, but is required for recent ones that lack sufficient consolidation. It is implied that recent memories are more susceptible to loss following hippocampal damage secondary to incomplete consolidated; therefore, a retrograde amnesia gradient is predicted.

Multiple Trace Theory was proposed as an alternative model (Nadel and Moscovitch, 1997). In contrast to SMSC, MTT proposes that the hippocampus has an important role in the retrieval of all episodic memories, including remote ones. In line with SMSC, MTT proposes that memories are encoded in hippocampal-neocortical networks, but that each reactivation results in a different trace in the hippocampus. It is presumed that hippocampal traces are contextual and rich in spatial details; on the other hand, cortical traces are presumed to be “semantic” and largely context free.

The central conflict between these theories is the role of the hippocampus in the retrieval of remote episode memories (Yassa and Reagh, 2013), episodic memories being defined as memories of specific events including times, places, and other contextual information that can be explicitly stated. Multiple Trace Theory predicts that hippocampal activation is required for episodic memories regardless of how old they are; therefore, there is no gradient for episodic memories.

As mentioned, SMSC predicts that a temporal graded retrograde amnesia manifests with MTL damage, given the assumption that older memories rely less on the hippocampus. Indeed, initial work with the amnestic patient H.M. demonstrated this; however, it later became clear that neuropsychiatric testing limitations of that era may not have adequately tested episodic memory (Corkin, 2002). In retrospect it was asserted that H.M. was unable to narrate even one event from his life that occurred at a specific time or place. On the other hand, patient E.P., who had more extensive MTL damage, retained highly detailed spatial memories of his childhood neighborhood were preserved (Stefanacci et al., 2000) arguing for a gradient. In turn, Rosenbaum et al., 2000 assessed patient K.C. who had extensive bilateral hippocampal damage but also to other regions. K.C. had severe retrograde non-gradient amnesia for autobiographical episodes but seemingly preserved detailed memory of his childhood neighborhood as well. However, upon closer inspection, it was discovered that he experienced great difficulty separating lures from actual landmarks in his neighborhood. Controls scored 45/48 and 46/48, but K.C. scored 15/48 (Rosenbaum et al., 2000). Basically neither theory can be fully proven or disproven due to conflicting clinical presentations and experimental results. Nevertheless, it should be made explicitly clear that memories of a neighborhood that were traversed thousands of times do not constitute episodic, a memory of a single event. This observation will be discussed in greater detail later.

## Hierarchical perspective

The organization of the brain recapitulates the hierarchical generation of sensations in the outside environment. In the world, or “external reality,” lower hierarchy levels are predicted by the upper levels. For example, the position of a ball can be determined by its velocity; velocity can be determined by acceleration, and acceleration can be determined by jerk (rate of change in acceleration)—over hierarchically nested timescales. This hierarchical causal structure is thought to be recapitulated in the hierarchical organization of the visual brain—for which there is an overwhelming amount of physiological and anatomical evidence. In this setting, the complexity of the brain increases from caudal to rostral—and implicitly to higher or deeper hierarchical levels of representation. The lower levels of the hierarchy are associated with sensory input, for example the primary auditory and visual cortex located in temporal and occipital regions respectively. These unimodal (meaning only one sense) levels are not directly interconnected to each other; however, they are connected to upper transmodal levels (Mesulam, 1998). Transmodal levels bind sensory input from multiple sensory modalities simultaneously. For example, words used in language are located in transmodal areas because they simultaneously hold visual and auditory associations (Mesulam, 1998). In transmodal areas, neuronal communication occurs within and between hierarchical levels. Relatively, lower levels represent information closer to sensory channels, while higher levels pertain to more abstract representations or “memories” that provide top-down predictions of lower-level expectations. In a simplified example, top level representations can hold broad contextual categories such as “office”; whereas, middle representations hold items such as chair, desk, cubicle, while lower representations contain graphical elements such as line, square, etc. Essentially, upper levels are composed of clusters of lower representations. In the office example, upper levels predict chairs, chairs predict chair legs, legs predict metal, plastic, or wood, etc. If descending predictions do not match upward sensory information, then “prediction errors” form which are passed upward to update and improve higher level expectations.

## The free energy principal and hierarchical models

By considering the brain as an organ of prediction or inference—based on hierarchical generative models—the work of Karl Friston explains certain aspects of memory, perception, and action (Friston, 2008, 2009, 2010; Kiebel et al., 2008). This work formalizes long-standing notions about the brain as generating hypotheses or explanations to explain the sensory input in terms of Bayesian inference. More specifically, it considers action and perception in terms of minimizing surprise or free energy. Surprise roughly translates to the difference between internal representations of reality vs. reality itself. The free energy principle suggests that adaptive systems (like the brain) that contend with a changing environment minimize surprise about sensory input. Mathematically, surprise is quantified by −ln *p*(*y*(*a*)|*m*), where *y*(*a*) is sensory input sampled under some action *a*, and *m* represents the hierarchical model that is entailed by the brain (Kiebel et al., 2008). If surprise is roughly the difference between internal representations of reality vs. reality itself, then the brain would have to know “true reality” in order to determine the difference. Obviously the brain lacks a “true reality map”; therefore, it relies on determining the lack of agreement or alignment between internal hierarchy levels, free energy—an upper limit on surprise (Friston, 2008, 2009). By aligning hierarchy levels, free energy is minimized. The concept of phonetic ambiguity may illustrate this more clearly. The verbal phrase “your children” sounds identical to “you’re children”. The listener minimizes surprise by aligning the broad categorical context, teacher speaking to parent or parent talking to child respectively; as a result, internal hierarchy agreement helps the brain determine what outside reality may be. A way to put this mathematically, free energy is always slightly greater than surprise and, under some simplifying assumptions, reduces to the amount of prediction error, distributed over the hierarchy (Friston, 2009). Therefore, minimizing free energy minimizes prediction error and implicitly surprise. Action is used to align the outside world to internal representations; thereby, decreasing free energy as well. For example, before lifting one’s hand, the internal representation of hand lifts and the brain notices a discrepancy from incoming sensory information depicting a resting hand vs. the internal representation of a lifted one. In order to align the outside world with the internal representation, action is used to lift the hand to match the internal representation. For the sake of simplicity however, action will not be discussed since the focus is memory, attention, and perception.

Within this framework, a working definition of attention is supplied. Attention is defined as the precision of, or confidence placed in, prediction errors. This means that attending to certain sensory streams or hierarchical representations corresponds to amplifying prediction errors in the appropriate sensory channels or hierarchical levels—so that these prediction errors have privileged access to higher hierarchical expectations. In other words, attention is the process of optimizing precision, through mechanisms such as synaptic gain amplification. Crucially, attention controls the relative influence of (top-down) prior expectations relative to (bottom-up) sensory evidence or prediction errors (Friston, 2009). Another way to explain this is that attention creates better communication between certain hierarchy levels, so that they can be aligned with maximum efficiency. In a hypothetical example, a soldier caught in a fire fight, would increase interconnectivity between lower hierarchical levels to better identify somatosensory information to increase chances of survival. Attention is placed on sensory processing at the expense of novel abstract thought. On the other hand, an author writing a book would presumably increase interconnectivity (attention) between mid to high hierarchy levels in order to convert abstract thought into words in a meaningful way, a process that does not require intense somatosensory attention.

In terms of neurophysiology, connections that pass prediction errors up the hierarchy are superficial pyramidal cells. Interestingly, these cells are primarily responsible for EEG signals that can be measured non-invasively. It is possible that oscillation patterns such as gamma, alpha, and delta waves are byproducts of attention on distinct levels of the hierarchy. On the other hand, sources of backward connections are largely the deep pyramidal cells which are believed to encode the expected causes of sensory states (Mumford, 1992; Friston, 2009).

## Introducing a centripetal hierarchical associative model of memory and perception

The boundary between perception and memory is not concrete (Cabeza, 2008; Cabeza et al., 2008; Friston, 2009, 2010). For example, there is currently a debate regarding the role of the hippocampus in memory and perception. Is the hippocampus primarily used in memory, perception, or both? It can be said that an individual perceives their memories while dreaming—a perception that requires no sensory input. Through the free energy framework the connections between memory and perception become apparent.

**Figure 1:**
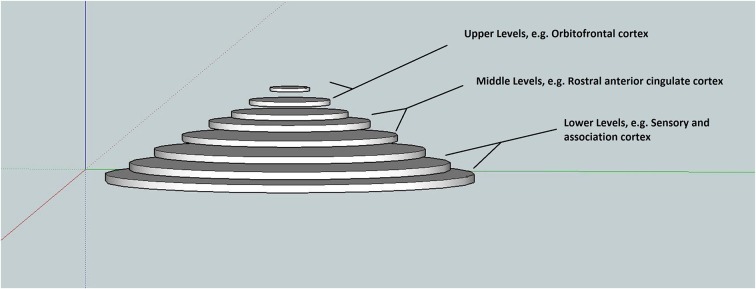
**An *aligned* memory residing simultaneously intra-hierarchically and inter-hierarchically**. Lower hierarchical levels contain rich sensory representations such as color, texture, smell. Notice that the frontal cortices are located at the top and center.

In this synthesis, a pyramidal hierarchical associative model of memory, based on (Friston, 2008), is presented that serves to facilitate a better understanding of hierarchical generative models and the constant predictive coding. The goal is to convey concepts intuitively and schematically—so that readers who are unfamiliar with the technical terminology appreciate the basic idea. Figure 1, depicts the associative hierarchy ranging from simple sensory elements such as “lines” up to abstract elements that simultaneously bind lower elements. Traversing up and down the hierarchical levels represents one (hierarchical) dimension of the model (Mesulam, 1998). Neuroanatomical considerations would place the top (or center) of the hierarchy in the frontal lobes e.g., orbitofrontal cortex (Kiebel et al., 2008), intermediate hierarchical levels for example in the rostral anterior cingulate cortex (Kiebel et al., 2008), and sensory areas at the lowest level (Mesulam, 1998; Kiebel et al., 2008). In this dimension, the MTL is located between the intermediate and upper levels. Given this framework, one may ask if it is correct treating the brain as homogenous or categorically uniform. If one defines the function of the whole brain as minimizing free energy, then it is possible. For example, the cerebellum plays a crucial role in generating predictions for perceptual and movement trajectories, consistent with lower levels.

The second aspect, within the model, considers memory *within* a hierarchy level; where a memory can occupy inter- and intra-hierarchical levels simultaneously. In other words, any given expectation or memory is bound within and between hierarchies—endowing memories with a distributed (between level) aspect and a representational (within level) content of greater or lesser sensory detail (Mesulam, 1998).

## Explicitly coupling memory with time

This model is consistent with other work that emphasizes time scales embedded within the hierarchy (Kiebel et al., 2008). The lowest level of the hierarchy corresponds to fast (seconds to milliseconds) fluctuations associated with sensory processing of a quickly changing sensorium. On the other hand, the highest levels encode slow contextual (conceptual) changes in the environment that can last weeks, months, and possibly a lifetime. In a sense, the lower levels have a faster “refresh rate” relative to higher levels. This faster rate may result from being closer to frequently changing sensory channels and being used by multiple higher representations. The framework proposed by Kiebel et al. (2008) also follows a neuroanatomical organization where hierarchical complexity increases moving from caudal to rostral.

According to this MCPC, the brain contains distributed aspects of the “same” memory, across all levels of the hierarchy, each level holding different levels of detail through different time spans. For example, a person looks at a picture of neighborhood for ten seconds, and then the picture is removed. Immediately afterwards, he can recall the picture with extreme detail; such as the number of cars, color of leaves, how many people are in the picture, and what color clothing they are wearing. This detail is represented in lower levels of hierarchy. As representations in the lower levels quickly decay, upper levels are called upon to retrieve the more abstract semantics associated with the scene, given a slower rate of decay. In fact, the phenomenon commonly described as “short-term memory” may be, in part, a manifestation of the dynamic nature of low-level expectations that have fast time constants and decay quickly (Mesulam, 1998). Indeed, the decay of expectations during hierarchical perceptual inference has been proposed as an explanation for binocular rivalry, where different percepts compete for dominance (Hohwy et al., 2008). Returning to the example, with the passage of time, the person may recall vague generalizations such as trees, cars, and people. A month later, when asked to recall the picture (assuming no intentional review or rehearsal was engaged) the person may give a more contextual account such as “a neighborhood street”. This phenomenon may be viewed as different versions of the same memory accessed at different hierarchical levels.

What the literature currently calls “source memory” may actually reflect lower levels of this hierarchy. For example, source memory can include contextual features such as color or visual elements such as location. The hierarchical (pyramidal) framework may also explain why patients with damage to high level associations cortex, located in the prefrontal cortex (PFC), exhibit mild to moderate impairments in detail rich retrieval (Mangels et al., 1996; Hwang et al., 2007; Shimamura, 2011). On the other hand, patients can have highly detailed false memories—presumably by activating inappropriate featural representations at lower levels in a top-down fashion. Similar mechanisms for false inference (e.g., hallucinations and delusions) in psychosis have been proposed that rest upon a loss of alignment—mediated by a failure of classical modulatory neurotransmission that is thought to encode attention (Adams et al., 2013). Put simply, false memories and hallucination and delusions may reflect an inability to attend to the appropriate hierarchy levels during retrieval and encoding respectively.

## A unified definition of memory and perception

The definitions of perception, encoding and retrieval are usually considered independently, but here they are collapsed into one simple definition: finding the maximum consilience among hierarchical levels through the process of attaining alignment. In technical terms, this entails the minimization of free energy (Friston, 2008, 2010; Henson and Gagnepain, 2010). In a sense, free energy can be viewed as a lack of alignment within hierarchical inference and its minimization corresponds to the creation of alignment. Encoding and perception will be considered synonymous for simplicity—and both involve a minimization of (precise) prediction errors throughout the hierarchy. Through this perspective, memory is not simply a recording of past experiences; instead, it becomes indivisible from perception and cognition. In a hypothetical example, a middle age man in therapy recalls how his college girlfriend broke up with him with the explanation that he was not attentive to her needs. Recalling this memory through a more experienced contextual understanding of the world, other memories come to mind. He also recalls that one of his buddies broke up with his girlfriend several weeks before the patient’s breakup. Suddenly, he realizes that his girlfriend may have ended the relationship because her secret crush was available, especially since they began dating shortly after their breakup. Here, a memory is recalled; however, it is altered when an overarching context explains seemingly unrelated events. Free energy is minimized and the memory will never be the same due to recontextualization.

The process of alignment can be guided from the top, bottom, or both (Friston, 2008, 2010). It may depend on which hierarchy level is inferred to have the strongest representation, such that the precision of the alignment empowers that particular level in guiding other levels. The weaker may “orient” to the stronger, but alignment is not a unilateral process: both the bottom and top levels “orient” each other until hierarchical consilience is achieved; however, the weakest level does most of the “orienting to”. In perception, the bottom is usually strongest and most stable due to precise sensory data from sensory channels; consequently, the top will orient to the bottom. In contrast, memory recall usually involves higher representations having more precision than bottom up influences—due to lower decay rates. Consequently, the bottom does most of the orienting to, relative to the top during the process of recall-alignment (or indeed dreaming). In computational terms, this process is labeled as pattern completion where a sparse cue may trigger a rich recollection via recurrent interactions between broad upper level representations and detailed lower level representations.

As outlined above, many roles have been attributed to the MTL. Many theories posit that the hippocampus, sitting on top of the hierarchy bind distant cortical regions during memory formation (Shimamura, 2011). MCPC diverges from this account and suggests that the MTL serves a conduit, through which supra-MTL levels interface with infra-MTL levels, via pathways that *skip* hierarchy levels; consequently the MTL or hippocampus resides in the middle of the hierarchy and not the apex. It should be made clear that direct communication between higher and lower hierarchical levels can occur without the MTL, via serial connectivity between consecutive levels in the cortex; however, it is supposed that the MTL is required for skipping levels and establishing a parallel connectivity between high and low levels. Within this framework, the MTL is just a relay; consequently, it is not responsible for binding event features *per se*; instead, the binding comes from above-hippocampal-hierarchy associations that access lower levels through the hippocampus and *vice versa*. The connections are reciprocal; thus allowing upper hierarchies to receive sensory information more quickly than the information ascending serially through all levels of the cortical hierarchy. In turn, the upper levels can send predictions to lower and mid-levels before being fully informed by serial bottom-up hierarchical inference. This “fast lane” approach may have evolved to overcome temporal limitations in species with expansive multimodal association areas, specifically in fast changing environments (Mesulam, 1998). Lower and middle levels that have been predicted by upper levels—via the MTL—enjoy top-down selection that contextualize the predictive coding through selecting appropriate processing channels (e.g., through top-down precision control or attention). This distinction can have a profound influence on what is attended to and remembered as the flood of sensory information serially sweeps up the hierarchy, a phenomenon that can be viewed from different perspectives. For example, it can be viewed as attention from the frontal cortices working through the hippocampus to “filter out” irrelevant information (Mesulam, 1998). From Friston’s perspective, it can represent how higher hierarchies predict the relative precision of prediction errors in lower levels to make predictive coding and learning more efficient. Psychologically, it may be construed in terms of (endogenous) attentional selection.

## A proposed role for the MTL

**Figure 2:**
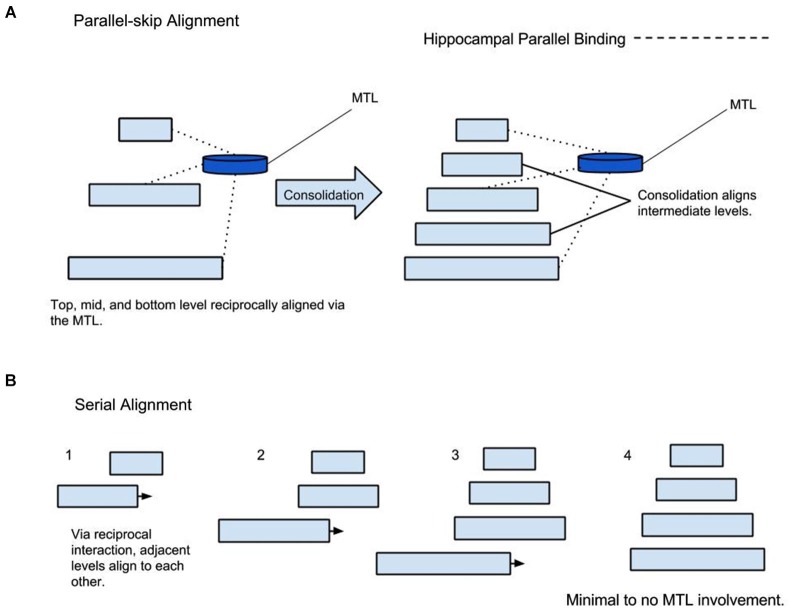
**Parallel and Serial Alignment. (A)** Parallel alignment of hierarchy levels mediated by the MTL followed by consolidation. **(B)** Demonstrating serial alignment of a completely consolidated representation without the MTL involvement.

It is proposed that two forms of alignment coexist, serial and parallel alignment (see Figure 2). In serial alignment, juxtaposed hierarchy levels reciprocally align each other. On the other hand, parallel alignment can occur between non-juxtaposed hierarchy levels via MTL mediated skipping or bridging—a theme that will be discussed in greater detail in the next section. Serial and parallel alignment may occur simultaneously (Mesulam, 1998). Indeed, such an arrangement has been demonstrated in lower visual association areas (Felleman and Van Essen, 1991; Mesulam, 1998). The concepts of different hierarchical time scales and interconnections between levels have been presented in order to present the next point: from a memory perspective, MTL mediated parallel alignment may afford lower levels a degree of temporal decay protection through descending coordination by upper levels. This may explain the requisite role of the hippocampus in conditions where conditioned and unconditioned stimuli are separated by a temporal gap but not where they overlap in time (Solomon et al., 1986; Clark and Squire, 1998). It is possible that the lack of temporal separation preserves the low-level representations from decay; reducing dependency on the MTL. On the other hand, after certain intervals, more support from higher levels, mediated via the MTL is required. With time, it is possible that this type of prediction/protection allows hierarchical consolidation to occur, a topic discussed later.

**Figure 3:**
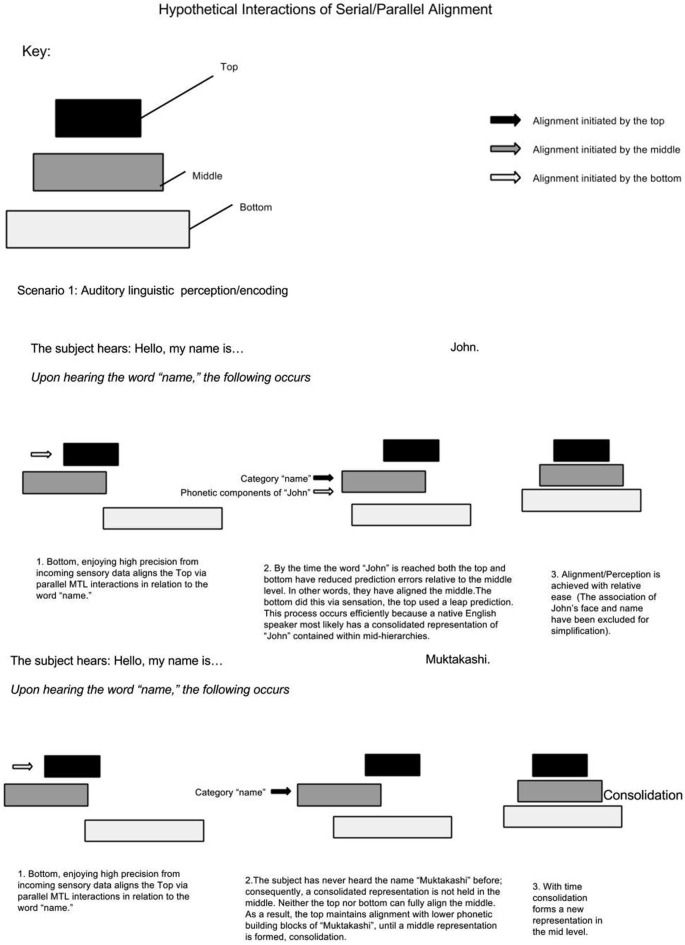
**Demonstrates alignment of consolidated vs. non-consolidated representations**.

Assuming MTL activity correlates with the number of non-adjacent representations bound, this model predicts less MTL activity when information within low-mid level hierarchies has a high ratio of consolidation/non-consolidation. For example, if a native English speaker meets a man who says, “Hello, my name is John”. Upon hearing the word “name”, supra-MTL hierarchies presumably predict associative areas holding names; as a result, the representation “John”, having enjoyed an extra boost (or greater precision), becomes bound via the MTL. It is presumed that the native English speaker has a consolidated representation of the name “John”. On the other hand, the native English speaker meets a man who says, “Hello, my name is Muktakashi”. It’s doubtful that the same associative area holds a consolidated representation of “Muktakashi”. Medial temporal lobe skipping must skip to lower phonetic-containing hierarchical levels; consequently, MTL activity increases because of more elementary or detailed “fragments” to bind (see Figure 3).

**Figure 4:**
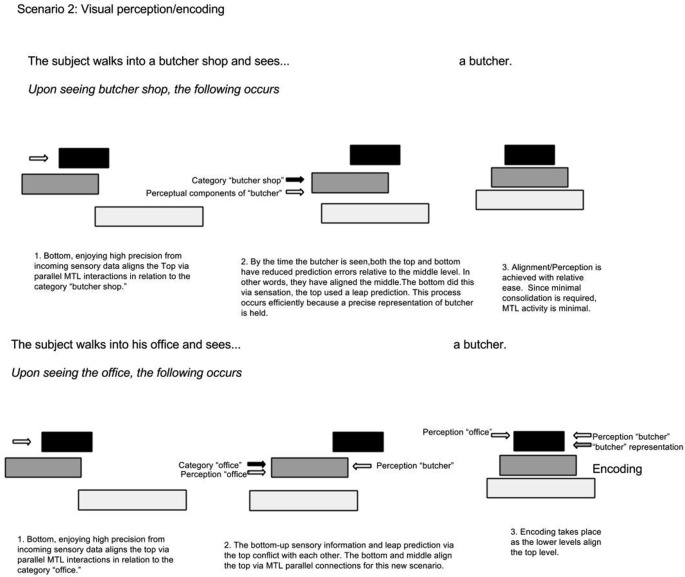
**In-context vs. out-context alignment.** The first scenario depicts a situation where the top level representation, containing the context, adequately predicts the situation. This contrasts to the second scenario where the top representation does not accurately predict the situation; as a result, it becomes updated by lower levels via the MTL.

The same phenomenon can explain increased MTL activity when something unexpected is perceived (Henson and Gagnepain, 2010). In the butcher shop example, it is predicted that MTL activity increases when an item (butcher) exists in an unlikely context (office), because these two representations are held on non-adjacent hierarchy levels and a consolidated context-representation of butcher-in-office does not exist. On the other hand, MTL activity is less if the butcher is seen at the butcher shop. In a simplified hypothetical example illustrated in Figure 4, upon walking into a butcher shop, lower levels align the top to the context of “butcher shop”; as a result, the top selects associations contained in the category “butcher shop”. This top level prediction is in agreement with serially ascending perceptual input of the butcher standing behind the counter; consequently there is minimal surprise and minimal need for MTL bridging and consolidation. In a different scenario, a computer programmer walks into his office. Prior to walking in, perceptual cues from lower levels activate top level context predictions via the MTL. In turn, top levels select categorical representations related to the office setting. Walking into his office, he sees a butcher. Immediately there is a clash between top-down contextual predictions and serially ascending perceptual content. Unlike the foreign name example, where the intermediate representation (of the foreign name) was missing; this time, the top level category representation is inadequate since the butcher has never been seen in the office context. This time, the MTL bridges the mid-level butcher representation to the upper level office context representations; consequently, the top level categorical association becomes realigned.

## Consolidation

In partial agreement with other theories, it is proposed that the *initial* skip-binding, or parallel alignment of hierarchy levels initiates an automatic process of structuring intermediate levels. In other words, the hierarchy levels between initially bound lower and upper hierarchy levels, for a specific memory, consolidate themselves for optimal *future* alignment after the perceptual or learning event has taken place. It is proposed that with sufficient consolidation, hierarchy levels can align via serial hierarchical connections vs. reliance on parallel alignment; consequently, the MTL does less work because serial alignment replaces skipping Figure 2. In many, studies different types of memories are tested including autobiographical, episodic, semantic, faces, famous faces, and events. In this framework, it is proposed that the ratio of consolidation to non-consolidation determines the degree of MTL involvement rather than the above classifications. It is proposed that consolidation is correlated to time and total number of alignments. Alignment can be initiated consciously or subconsciously via bottom-up, top-down, and affective activation. Bottom-up alignment can include walking in one’s neighborhood every day. Top down could be repetitive rehearsal such as saying a prayer every morning. Affective induced alignment may manifest as a trauma playing over and over again secondary to emotional value. Under this schema, one can imagine that a unique episode re-aligns relatively less often than walking through one’s neighborhood. Famous faces and specific events may happen more frequently; however, it is highly idiosyncratic. On the other hand, walking through one’s neighborhood and semantic knowledge are frequently realigned events; therefore, they would have the highest consolidation/non-consolidation ratio. Linguistic phrases would be expected to have high consolidation since they are produced and discerned frequently.

## Consolidation in relation to sleep and default mode network activity

Upper to mid-level consolidation may occur during sleep and default mode network activity, when attention is not fixated on lower hierarchy levels. Indeed, the essential role of sleep has been discussed in terms of optimizing alignment in hierarchical generative models through minimizing complexity (redundancy). This provides a simple perspective on the neurochemistry of sleep and the sensory gating associated with dreaming (Hobson and Friston, 2012)—and introspection (reflection) during default mode network activity (Carhart-Harris and Friston, 2010).

## The formation of non-declarative memories via bottom-up alignment

Based on what has been proposed so far, it would be expected that long term memories cannot form without the MTL; however, clinical experience shows otherwise. Patients with bilateral MTL damage have been shown to navigate in new houses or neighborhoods—after some time—but without the ability to declare how they navigated (Stefanacci et al., 2000). To understand how “non-declarative” memories form without the temporal protection of higher hierarchies, the mechanism of temporal protection must be considered: in essence, higher levels maintain a specific alignment pattern in lower hierarchical levels, so that consolidation occurs. The same phenomenon can be recreated if input from lower hierarchy levels—namely, perception—is kept sufficiently constant. In other words, if someone with MTL damage navigates an environment long enough, the lower hierarchy levels will be repeated a sufficient number of times to consolidate without requiring higher level temporal protection or contextual guidance. The next question is why the patient cannot access or declare their memory. To resolve this paradox, it is proposed that the alignment process is dissociated from upper hierarchy levels. Lacking connection to higher levels will prevent this memory from being consciously accessed or declared, a dissociated memory. In other words, a disconnection between high and low level representations precludes the formation of more abstract memories that presumably constitute the content of access consciousness. In terms of predictive coding, this is a natural consequence of removing bottom-up prediction errors that are necessary for the formation and learning of concepts in terms of high level expectations.

## Relating the centripetal hierarchical framework to perception

As touched upon earlier, perception can be explained by the centripetal hierarchical associative model as well. Driving information straight from sensory channels, the bottom levels may become relatively stronger than upper hierarchical levels. In a sense, this becomes the reverse of memory, where top levels are *usually* stronger than the bottom. As opposed to “recall”, alignment in the opposite direction is the phenomenon of (exogenous attentional) orientation; consequently, detail rich components orient to a broad context, the upper hierarchy levels. This is consistent with attentional adjustments to the precision of prediction errors, through adjusting neuromodulatory or cortical gain selectively at different hierarchical levels. In recollection, introspection, and sleep there is an attenuation of sensory or low-level precision; while during alert waking perception, there is the converse increase in sensory precision: see (Feldman and Friston, 2010) and (Brown et al., 2013) for a simulations of this in terms of predictive coding, biased competition and classical psychophysics experiments.

Some recent literature discusses a possible role of the MTL in perception. From the bottom-up perspective, skipping may allow an irrelevant sea of intermediate representations to be bypassed in order to reach appropriate upper level processing. This may be adaptive in situations where speed is essential. In studies looking at monkeys with perirhinal cortex (PRC) damage (located within the MTL), there was a disruption in simultaneous and zero-delay matching-to-sample of perceptually similar object stimuli (Eacott et al., 1994) and impairment in discrimination of simultaneously presented objects that share a high number of visual features—or objects that are shown from different points of view (Buckley et al., 2001; Lee et al., 2012). Another study, looking at amnestic patients with MTL damage, found that the hippocampus is essential for discriminating scenes with a high degree of overlap, while the PRC is required for discriminating scenes and faces with high overlap of features (Lee et al., 2005). Interestingly, these deficits existed in the context of intact perception of simple visual features such as color and size (Lee et al., 2012). As discussed above, upper hierarchy levels may need to “inform” lower levels to resolve intra-hierarchical conflict or ambiguity; however, such processing would not be necessary for simple visual features.

Recalling patient K.C., who had detailed memories of his neighborhood but inability to tell lures from actual landmarks, a similarity emerges with the above experimental findings. Medial temporal lobe damaged appears to impair both discrimination of simultaneously presented images that share visual features and memories from the distant past vs. images that share visual features. Here it is proposed that upper hierarchy levels are bridged via the MTL to resolved conflict in non-adjacent lower levels regardless of whether it is a distant memory or simultaneously presented image.

## Predictive, interactive multiple memory systems and MCPC

The Predictive, Interactive Multiple Memory Systems (PIMMS) model most closely resembles the framework of MCPC. A major emphasis of PIMMS is that memory encoding and retrieval occurs, via reciprocal interconnections between hierarchical levels with the aim of minimizing prediction error, the same as MCPC (Henson and Gagnepain, 2010). In order to convey important concepts, PIMMS divides memory hierarchies into three types/levels: episodic, semantic, and perceptual. Instead of looking at the MTL as one functional unit, PIMMS looks at certain components of the MTL and other regions in relation memory types. They designate the hippocampus and PRC, both components of the MTL, as key parts of the episodic and semantic systems respectively. Predictive, Interactive Multiple Memory Systems states that the key function of the hippocampus is to optimize the mutual predictability between items and context. It states that mutual predictability corresponds to the joint probability of predicting an item from a context and predicting a context from an item. For example, “representations of the current context is used by the hippocampus to predict items that are likely to appear in that context” (Henson and Gagnepain, 2010). This is closely related to the concept of alignment; especially when canst in terms of top-down predictions of context and attentional (or precision based) selection of lower-level representations. Furthermore, Predictive, interactive multiple memory systems model is one of the first to describe how predictions can be framed within the hierarchical interactive memory system view (Henson and Gagnepain, 2010). PIMMS also distinguishes an acute from delayed component of encoding, analogous to encoding and consolidation respectively; although, the mechanics of consolidation are not explored in contrast to MCPC.

Divergent to MCPC, PIMMS places the hippocampus at the apex of the hierarchy; thus, the hippocampus is defined as the component doing the predicting. As discussed already, MCPC defines the hippocampus as a mid-level conduit for parallel connectivity with upper hierarchies; therefore, upper hierarchies are doing the predicting. Another contrast is that this MCPC looks at the MTL as a whole rather than dividing it. The question of whether the MTL serves one function vs. multiple is dependent on the definition of “function”. Medialtemporal-lobe Conduit for Parallel Connectivity defines this function as bridging upper to lower representations to allow parallel processing; therefore, it is possible to view the MTL as a unit serving one function. By placing the hippocampus in the middle of the hierarchy, MCPC is able to distinguish between two types of hierarchical alignment: serial vs. parallel, a distinction that can explain some subtleties of consolidation.

## Revisiting SMSC and MTT

Going back to SMSC and MTT theories of memory, they are both partially in agreement with MCPC. According to MCPC, with sufficient re-alignments, a memory becomes sufficiently consolidated thus requiring minimal MTL activity, at least in most recall tasks (e.g., conditions with minimal conflict). On the other hand, in episodic memories that do not align often and lack sufficient consolidation, MTL bridging becomes mandatory for detailed recall regardless of memory age. Medialtemporal-lobe Conduit for Parallel Connectivity explicitly explores the difference between detail rich episodic memories vs. detail recollection of a childhood neighborhood. The latter enjoys frequent bottom-up alignment thus increasing consolidation rates. Furthermore, it is possible that childhood memories are encoded with higher precision, given that language accents form after a certain age; however, this is out of the scope of this paper. According to MCPC bottom-up alignment may occur independent of MTL function. In addition, MCPC predicts that MTL bridging is required for certain tasks regardless of full memory consolidation. Such tasks include lure discrimination where lower levels need direct access to high level predictions to reduce conflict. In other words, MTL bridging adds additional dimensions of recollection, ones that become visible through a hierarchical free energy perspective. These additional dimensions are not limited to recall, but can also involve recontextualization as in the case of the patient recalling a college breakup. In summary, MTL function can occur during recollection of fully consolidated memory; however, it is not vital for basic recollection. This finding may reconcile MTT’s evidence that medial temporal fMRI activity was equally predictive of recent and remote memory retrieval (Nadel and Moscovitch, 1997) with SMSC’s observation that amnesiacs can have rich recall of childhood neighbors. However, great caution must be exercised with studies looking at MTL activation since consolidation for other memories may occur in parallel with the task at hand.

## Predictions of MCPC

Looking at prior memory experiments, it is clear that testing memory can prove difficult. One difficulty is that terminology such as “episodic” or “semantic” have a degree of ambiguity. Another problem is that memory and perception can be highly idiosyncratic. One method to test certain predictions of MCPC is correlating MTL activity to new language acquisition. The speech of patient H.M., K.C., and E.P. was relatively spared; therefore, MTL is not essential for well consolidated speech. However, MCPC predicts that a subtle gradient may exist for secondary languages that have not enjoyed the same number of alignments with high precision encoding.

The study can observe MTL activity in 3 groups. In the first group, MTL activity is assessed while speaking the native language. In the second group, MTL activity is assessed through time as subjects learn a foreign language. It is predicted that MTL activity in the second group will be higher than the first, will decrease over time, and will not drop below the first. The third group will consist of fluent bilinguals who acquired the second language after age eleven. It is predicted that the third group will have MTL activity between the first 2 groups during specific speech acts.

## Conclusion

Ultimately, the main function of MCPC is to view the MTL through the free energy paradigm as opposed to a framework where the connections between perception, cognition, and memory are not apparent. Through the free energy paradigm perception, memory, cognition, action, and attention are interdependent processes that function, on a hierarchy, to lower free energy. This perspective can resolve contradictions in research findings such as preserved non-declarative memory in MTL damaged patients, detailed childhood memories of locations but not episodes, inability to distinguish objects that share common features with MTL damage, and continued MTL activity on presumably consolidated memories.

## Future directions

One major element that MCPC lacks is how memory, perception, and action interact with emotion. Indeed, research demonstrates a great deal of interaction between emotion, perception, and memory (Pourtois et al., 2012). It is certainly not a coincidence that the amygdala, a major affective center, is located in the MTL. Further work may aim to integrate affective and cognitive components to discover a more thorough understanding of this topic. By integrating the role of emotions in minimizing free energy, a more complete view of brain functioning may form.

Another application of the free energy hierarchical perspective can be in child development. How does development correlate to hierarchical development? For example, when infants gain the ability to distinguish self from others, does that correlate to the formations of contextual representations of self/others within upper hierarchies? It may even explain why early traumas profoundly influence patients for a lifetime or why accents form when language is acquired after a certain age. The applications certainly seem limitless at this point.

## Conflict of interest statement

Psychiatry Private Practice Brian Mozaffari M.D., Inc. The authors declare that the research was conducted in the absence of any commercial or financial relationships that could be construed as a potential conflict of interest.
